# Incidence and Progression of Diabetic Retinopathy in American Indian and Alaska Native Individuals Served by the Indian Health Service, 2015-2019

**DOI:** 10.1001/jamaophthalmol.2023.0167

**Published:** 2023-03-09

**Authors:** Stephanie J. Fonda, Sven-Erik Bursell, Drew G. Lewis, Dawn Clary, Dara Shahon, Jerry Cavallerano

**Affiliations:** 1 Estenda Solutions Inc, Wayne, Pennsylvania; 2 Phoenix Indian Medical Center, Phoenix, Arizona; 3 Beetham Eye Institute, Joslin Diabetes Center, Boston, Massachusetts; 4 Department of Ophthalmology, Harvard Medical School, Boston, Massachusetts

## Abstract

**Importance:**

Estimates of diabetic retinopathy (DR) incidence and progression in American Indian and Alaska Native individuals are based on data from before 1992 and may not be informative for strategizing resources and practice patterns.

**Objective:**

To examine incidence and progression of DR in American Indian and Alaska Native individuals.

**Design, Setting, and Participants:**

This was a retrospective cohort study conducted from January 1, 2015, to December 31, 2019, and included adults with diabetes and no evidence of DR or mild nonproliferative DR (NPDR) in 2015 who were reexamined at least 1 time during the 2016 to 2019 period. The study setting was the Indian Health Service (IHS) teleophthalmology program for diabetic eye disease.

**Exposure:**

Development of new DR or worsening of mild NPDR in American Indian and Alaska Native individuals with diabetes.

**Main Outcomes and Measures:**

Outcomes were any increase in DR, 2 or more (2+) step increases, and overall change in DR severity. Patients were evaluated with nonmydriatic ultra-widefield imaging (UWFI) or nonmydriatic fundus photography (NMFP). Standard risk factors were included.

**Results:**

The total cohort of 8374 individuals had a mean (SD) age of 53.2 (12.2) years and a mean (SD) hemoglobin A_1c_ level of 8.3% (2.2%) in 2015, and 4775 were female (57.0%). Of patients with no DR in 2015, 18.0% (1280 of 7097) had mild NPDR or worse in 2016 to 2019, and 0.1% (10 of 7097) had PDR. The incidence rate from no DR to any DR was 69.6 cases per 1000 person-years at risk. A total of 6.2% of participants (441 of 7097) progressed from no DR to moderate NPDR or worse (ie, 2+ step increase; 24.0 cases per 1000 person-years at risk). Of patients with mild NPDR in 2015, 27.2% (347 of 1277) progressed to moderate NPDR or worse in 2016 to 2019, and 2.3% (30 of 1277) progressed to severe NPDR or worse (ie, 2+ step progression). Incidence and progression were associated with expected risk factors and evaluation with UWFI.

**Conclusions and Relevance:**

In this cohort study, the estimates of DR incidence and progression were lower than those previously reported for American Indian and Alaska Native individuals. The results suggest extending the time between DR re-evaluations for certain patients in this population, if follow-up compliance and visual acuity outcomes are not jeopardized.

## Key Points

**Question:**

What are current rates of incidence and progression for diabetic retinopathy (DR) in American Indian and Alaska Native individuals?

**Findings:**

In this cohort study of 8374 American Indian and Alaska Native individuals evaluated in 2015 and again in 2016 to 2019 by the Indian Health Service (IHS) teleophthalmology program, cumulative incidence of DR (any level) was 18%, and 27% of patients with mild nonproliferative DR (NPDR) progressed to moderate NPDR or worse, lower than previous reports.

**Meaning:**

These estimates suggest the viability of extending follow-up intervals for retinopathy assessment in IHS patients with no DR or mild NPDR if follow-up compliance is not jeopardized.

## Teaser

This cohort study investigates the rates of incidence and progression for diabetic retinopathy in American Indian and Alaska Native individuals.

## Introduction

Although the rate of increase has declined recently,^1^ diabetes prevalence in American Indian and Alaska Native individuals remains higher than in other race and ethnic groups in the US, with 14.7% of American Indian and Alaska Native individuals having been diagnosed with diabetes.^2^ The Centers for Disease Control and Prevention predicts that one-half of American Indian and Alaska Native individuals born in 2000 will develop diabetes some time in their lives.^3^ Additionally, an analysis of 1990 to 1998 Indian Health Service (IHS) outpatient data found that diabetes is being diagnosed at younger ages in American Indian and Alaska Native individuals.^4^ Longer duration could mean greater likelihood of diabetes-associated complications. In this vein, between 2015 and 2050, the number of people who are blind is projected to double,^5^ and diabetic retinopathy (DR) will likely be a major contributor because DR remains a leading cause of preventable blindness in US adults.^6^

In this context of disease burden, it is important to understand the prevalence and incidence of diabetes complications to allocate surveillance programs and specialty services appropriately. Recent publications suggest that diabetic eye disease prevalence has declined in American Indian and Alaska Native patients of the US IHS.^7,8^ However, estimates of DR incidence in American Indian and Alaska Native patients are not current; instead, they based on data from before 1992.^9,10,11,12^ This study estimates recent cumulative incidence, incidence rates, and progression of DR in American Indian and Alaska Native patients served by this IHS primary care–based teleophthalmology program.

## Methods

### Setting and Study Population

This was a retrospective cohort study using deidentified medical record data obtained during routine clinical operations of the IHS teleophthalmology program at 75 primary care clinics distributed among 20 states. The IHS serves enrolled members of federally recognized tribes. The study was reviewed and approved by the IHS institutional review board at Phoenix Indian Medical Center under the exempt process. Written informed consent from participants was not required or obtained.

Details regarding the teleophthalmology program’s origins, protocols, distribution, and outcomes have been previously described.^13^ Briefly, the program evaluates patients from participating primary care clinics. It is a validated American Telemedicine Association Category 3 program and its graders identify the Early Treatment Diabetic Retinopathy Study (ETDRS)–defined clinical levels of DR and diabetic macular edema (DME) severity.^13,14,15^ Graders are certified and licensed optometrists who render a diagnosis using standardized protocols. The program currently recommends that patients receive annual DR examinations.

Before selecting the analytic cohort for this study, we defined a baseline period of January 1, 2015, to December 31, 2015, and a follow-up period of January 1, 2016, to December 31, 2019. Eligible patients had at least 1 IHS teleophthalmology examination with the program in both periods. Additionally, eligible patients were 20 years or older and had no evidence of DR or had mild nonproliferative DR (NPDR; ETDRS levels 10, 14, 15, 20) in the baseline period. Patients with severe/very severe NPDR (ETDRS levels 53 a-e), proliferative DR (PDR; ETDRS levels 61, 65, 71, 75, 81, 85), and/or any level of DME are referred out of the teleophthalmology program to specialty eye care; therefore, these patients were excluded. Referral recommendations of patients with moderate NPDR (ETDRS levels 35, 43, 47) are dependent on risk factors; therefore, these patients were also excluded. This study followed the Strengthening the Reporting of Observational Studies in Epidemiology (STROBE) reporting guidelines.

### Measures

#### Outcomes in the Follow-up Period

The IHS teleophthalmology program uses 2 configurations of commercially available technology for image acquisition to assess DR severity level. The first configuration uses a low-illumination, nonmydriatic fundus photography (NMFP) digital imaging system (Topcon NW6S [Topcon Medical Systems]) with a custom digital camera back (Megavision Retinal Image Capture).^16^ Three nonsimultaneous stereo-pair 45° images from different retinal regions and 2 nonsimultaneous stereo-pair 30° digital images of the optic disc and the macula from the retina of each eye are obtained, for an approximate total retinal coverage of 90° to 135°. One external image of each eye is also obtained. NMFP showed substantial agreement with ETDRS controls for diagnosis of DR severity level (unweighted κ = 0.81; 95% CI, 0.73-0.89).^16^ The second configuration is nonmydriatic ultra-widefield imaging (UWFI) scanning laser ophthalmoscopy (SLO) (Daytona [Optos]). The UWFI protocol includes nonsimultaneous stereo-pair 200° images from each eye, centered on the macula. Previous research has shown that UWFI agrees perfectly with ETDRS photography in 84% of cases and agrees within 1 level of severity in 91% of cases (unweighted κ = 0.79).^17^ UWFI is the dominant configuration this program uses.

The grading outcomes were no evidence of DR, mild NPDR, moderate NPDR, severe/very severe NPDR, PDR, or ungradable. Level of DR at any 1 imaging encounter was defined by the more severely affected eye. If 1 eye was ungradable, the diagnosis for the other eye was used. If a patient received more than 1 teleophthalmology examination during the follow-up period, their maximum diagnosis was used in this analysis.

This study measured incidence and progression as follows: (1) any increase in level of DR; (2) occurrence of a 2 or more (2+) step increase; and (3) DR severity level. For patients with no evidence of DR at baseline, any increase in level meant mild NPDR or worse was found at follow-up, and a 2+ step increase meant moderate NPDR or worse was found. For patients with mild NPDR at baseline, any increase in level meant moderate NPDR or worse was found, and a 2+ step increase meant severe NPDR or worse was found. Severity levels at follow-up included patients who regressed from mild NPDR to no DR, but regression was not explored.

#### Background Variables

The IHS teleophthalmology program records patient demographics (age, sex [self-reported]) and known DR risk factors in templates used by the imagers and graders, taking data from the IHS electronic medical record patient summary. Risk factors recorded include glycosylated hemoglobin A_1c_ (HbA_1c_) level, diabetes therapy, duration of diabetes (but not diabetes type), hypertension, cardiovascular disease, hypercholesterolemia, peripheral neuropathy, and nephropathy. The program also records the clinic where the imaging occurred and whether UWFI or NMFP was used. We created measures indicating whether UWFI (vs NMFP) was used at baseline only, follow-up only, both examinations, or never. IHS administrative areas (derived from clinic addresses) are shown to describe the geographical distribution of the cohort.

### Statistical Analysis

The analysis calculated descriptive statistics of the selected cohort’s baseline characteristics and the imaging modalities patients were examined with over time. The analysis also calculated descriptive statistics for the patients who did not have a follow-up examination for comparison with the selected cohort. We compared the groups with *t* tests and χ^2^ tests.

The analysis next calculated cumulative incidence, cumulative progression, incidence rate, and progression rate. The rates equaled the number of new or worse cases of DR identified during the 2016 to 2019 period divided by total person-years (PY) at risk.^18^ PY contributed by a patient were truncated at the date of their examination that identified new or worsening DR. If the patient did not develop new DR or have worsening or progression of their DR, the PY they contributed was truncated at the date of their last examination in the follow-up period (≤4 years).

To estimate net associations between background characteristics and outcomes, the analyses conducted separate multivariable robust Poisson regressions. The dependent variables were as follows: (1) any new DR in patients with no DR at baseline, (2) occurrence of a 2+ step increase in DR for patients with no DR at baseline, and (3) any progression of DR in patients with mild NPDR at baseline. Variables representing imaging modality assessed whether UWFI increased detection of worsening disease net of other factors. Analyses obtained the robust SEs to calculate the CIs and 2-sided *P* values.^19^ *P* values < .05 were considered statistically significant. A model for a 2+ step increase in DR from baseline mild NPDR was not estimated due to the small number of patients in this category.

Descriptive statistics were performed using SAS software, version 9.4 (SAS Institute). Incidence rates and robust Poisson regressions were calculated using R software, version 4.1.2 (R Core Team).^20^

## Results

### Patient Characteristics and Imaging Modality

The number of patients evaluated by the program in the baseline year who were 20 years or older and had no evidence of DR or mild NPDR at that examination was 13 694 (Table 1). Of these patients, 8374 (61.2%; mean [SD] age of 53.2 [12.2] years; 4775 females [57.0%]; 3599 males [43.0%]) had at least 1 examination during the follow-up period. Mean (SD) time from the baseline examination to the first follow-up examination was 20.7 (9.5) months. A total of 4581 of 8374 patients (54.7%) who were followed up had 2 or more examinations during the follow-up period.

**Table 1.** Characteristics of the Selected Cohort vs Patients With No Follow-up Teleophthalmology Examination

Characteristic	No. (%)^a^		*P* value
	Selected (n = 8374)	No follow-up (n = 5320)	
No DR at baseline	7097 (84.8)	4493 (84.5)	.64
Mild NPDR at baseline	1277 (15.3)	827 (15.6)	
Age, mean (SD), y	53.2 (12.2)	52.8 (13.7)	.08
Sex (self-report)			
Male	3599 (43.0)	2260 (42.5)	.57
Female	4775 (57.0)	3060 (57.5)	
Diabetes duration, mean (SD),^b^ y	8.6 (7.4)	8.8 (8.1)	.27
Diabetes duration categories, y			
<1	689 (8.2)	496 (9.3)	<.001
1-5	2396 (28.6)	1381 (26.0)	
6-10	1791 (21.4)	904 (17.0)	
11-15	1236 (14.8)	655 (12.3)	
>15	1143 (13.7)	752 (14.1)	
Not specified	1119 (13.4)	1132 (21.3)	
HbA_1c_, mean (SD),^b^ %	8.3 (2.2)	8.2 (2.3)	.01
Missing a recent HbA_1c_ value			
No	6789 (81.1)	4265 (80.2)	.19
Yes	1585 (18.9)	1055 (19.8)	
Diabetes therapy			
Diet only	618 (7.4)	498 (9.4)	<.001
Oral medications only	4401 (52.6)	2483 (46.7)	
Insulin only	722 (8.6)	585 (11.0)	
Oral medications and insulin	1557 (18.6)	795 (14.9)	
Not specified	1076 (12.8)	959 (18.0)	
**Other patient diagnoses**			
Hypercholesterolemia			
Not present	5643 (67.4)	3906 (73.4)	<.001
Present	2731 (32.6)	1414 (26.6)	
Cardiovascular disease			
Not present	7837 (93.6)	4963 (93.3)	.49
Present	537 (6.4)	357 (6.7)	
Hypertension			
Not present	3815 (45.6)	2903 (55.6)	<.001
Present	4559 (54.4)	2417 (45.4)	
Peripheral neuropathy			
Not present	7866 (93.9)	4971 (93.4)	.24
Present	508 (6.1)	349 (6.6)	
Nephropathy			
Not present	7982 (95.3)	5108 (96.0)	.05
Present	392 (4.7)	212 (4.0)	
IHS office/region			
Alaska (5 clinics)	12 (0.1)	72 (1.3)	<.001
Albuquerque (5 clinics)	342 (4.1)	574 (10.8)	
Bemidji (5 clinics)	141 (1.7)	272 (5.1)	
Billings (7 clinics)	177 (2.1)	216 (4.1)	
Great Plains (6 clinics)	331 (4.0)	438 (8.2)	
Nashville (5 clinics)	155 (1.9)	45 (0.9)	
Navajo (9 clinics)	2402 (28.7)	1479 (27.8)	
Oklahoma City (13 clinics)	1437 (17.2)	793 (14.9)	
Phoenix (10 clinics)	2594 (31.0)	855 (16.1)	
Portland (12 clinics)	443 (5.3)	390 (7.3)	
Tucson (3 clinics)	340 (4.1)	186 (3.5)	
Imaging technology			
NMFP			
Both examinations	734 (8.8)	NA	NA
First examination, UWFI second examination	1839 (22.0)		
UWFI			
First examination, NMFP second examination	476 (5.7)	NA	NA
Both examinations	5325 (63.6)		

Abbreviations: DR, diabetic retinopathy; HbA_1c_, hemoglobin A_1c_; IHS, Indian Health Service; NA, not applicable; NMFP, nonmydriatic fundus photography; NPDR, nonproliferative DR; UWFI, nonmydriatic ultra-widefield imaging.

SI conversion factor: To convert HbA_1c_ to proportion of total Hb, multiply by 0.01.

^a^ Percentages may not sum to 100% due to rounding.

^b^ Excludes missing data (no imputations used).

In 2015, the mean (SD) HbA_1c_ level of the analyzed cohort was 8.3% (2.2%; to convert HbA_1c_ to proportion of total Hb, multiply by 0.01) (Table 1). The mean (SD) duration of diabetes was 8.6 (7.4) years, and 4401 of 8374 patients (52.6%) were managing their diabetes with oral medications only. Hypercholesterolemia (2731 [32.6%]) and hypertension (4559 [54.4%]) were the most common risk factors. A total of 53.4% of patients (2436 of 4559) in the analytic cohort with diagnosed hypertension were taking blood pressure–lowering medications (percentage not shown in Table 1). The Phoenix, Navajo, and Oklahoma City IHS areas imaged the most patients, with 2594 (31.0%), 2402 (28.7%), and 1437 (17.2%) being imaged, respectively. A total of 5325 of 8374 patients (63.6%) were imaged with UWFI both times, 1839 (22.0%) were imaged with UWFI at follow-up only, and 734 (8.8%) were imaged with NMFP for both examinations.

The selected cohort characteristics were not significantly different from patients who did not have a follow-up examination, except that proportionally fewer of them were missing information about their diabetes duration and diabetes therapy, their mean HbA_1c_ level was slightly higher (mean [SD], 8.3% [2.2%] vs 8.2% [2.3%]), and proportionally more of them had hypercholesterolemia (2731 of 8374 [32.6%] vs 1414 of 5320 [26.6%]), hypertension (4559 [54.4%] vs 2417 [45.4%]), or nephropathy (392 [4.7%] vs 212 [4.0%]) (Table 1).

### Incidence and Progression

Of patients with no evidence of DR at baseline, 1280 of 7097 (18.0%) had some level of DR at follow-up, for an incidence rate of 69.6 cases per 1000 PY (Table 2). Of the new DR found, 839 of 1280 cases (65.5%) were mild NPDR. Cumulative incidence of PDR was 0.1% (10 of 7097), for an incidence rate of 0.5 cases per 1000 PY. Of patients with no evidence of DR at baseline, 441 of 7097 (6.2%) had a 2+ step increase in DR over time (24.0 cases per 1000 PY).

**Table 2.** Incidence or Progression for Patients With No Diabetic Retinopathy (DR) or Mild Nonproliferative DR (NPDR) at Baseline: Frequencies, Percentages, and Rates

Baseline status	Follow-up status							
	Any increase	2+ Step increase	Severity level					
			No evidence	NPDR			PDR	Ungradable
				Mild	Moderate	Severe/very severe		
**No DR (n = 7097)** ^a^								
No. (%)	1280 (18.0)	441 (6.2)	5582 (78.7)	839 (11.8)	431 (6.1)	0 (0)	10 (.14)	235 (3.3)
Cases/1000 PY	69.6	24.0	NA	45.6	23.4	0	.5	NA
Cases/1000 PY (95% CI)	(65.8-73.5)	(21.8-26.3)	NA	(42.6-48.8)	(21.3-25.7)	0	(.3-1.0)	NA
**Mild NPDR (n = 1277)** ^b^								
No. (%)	347 (27.2)	30 (2.3)	427 (33.4)	437 (34.2)	317 (24.8)	2 (0.16)	28 (2.2)	66 (5.2)
Cases/1000 PY	111.7	9.7	NA	NA	102.0	.6	9.0	NA
Cases/1000 PY (95% CI)	(100.2-124.0)	(6.5-13.8)	NA	NA	(91.1-113.9)	(0.1-2.3)	(6.0-13.0)	NA

Abbreviations: NA, not applicable; PY, person-years at risk.

^a^ For the cohort with no DR at baseline, total PY used for any increase and severity level was 18398.9, and total PY for 2+ step increase was 18966.2.

^b^ For the cohort with mild NPDR at baseline, total PY used for any increase and severity level was 3107.7, and total PY for 2+ step increase was 3321.9.

A total of 347 of 1277 patients (27.2%) with mild NPDR at baseline developed a more severe DR level in the follow-up period, for an incidence rate of 111.7 cases per 1000 PY. A 2+ step increase in DR occurred for 2.3% of these patients (30 of 1277). Regarding DR severity level, cumulative incidences of severe/very severe NPDR and PDR were 0.2% (2 of 1277) and 2.2% (28 of 1277), respectively, for incidence rates of 0.6 and 9.0 cases per 1000 PY, respectively.

### Patient Characteristics and DR Outcomes

Characteristics associated with any DR incidence as well as occurrence of a 2+ step increase were longer diabetes duration (>15 y, any DR: risk ratio [RR], 2.0, 95% CI, 1.7-2.4; *P* < .001; 2+ step: RR, 3.2; 95% CI, 2.3-4.4; *P* < .001), higher HbA_1c_ level (any DR: RR, 1.1; 95% CI, 1.1-1.2; *P* < .001; 2+ step: RR, 1.3; 95% CI, 1.2-1.3; *P* < .001), and diabetes therapy, particularly insulin use alone (any DR: RR, 2.1; 95% CI, 1.5-2.9; *P* < .001; 2+ step: RR, 4.5; 95% CI, 1.8-11.2; *P* = .001) or with oral medications (any DR: RR, 2.2; 95% CI, 1.6-3.0; *P* < .001; 2+ step: RR, 4.5; 95% CI, 1.8-11.1; *P* = .001) (Table 3). For example, compared with patients receiving diet therapy alone, patients taking both oral medications and insulin had 4.5 times the rate of a 2+ step increase in DR. Notable characteristics associated with any progression from mild NPDR were longer duration of diabetes (>15 y, RR, 1.8; 95% CI, 1.2-2.5; *P* = .002), higher HbA_1c_ level (RR, 1.1; 95% CI, 1.0-1.1; *P* < .001), and presence of peripheral neuropathy (RR, 1.5; 95% CI, 1.2-2.0; *P* = .001).

**Table 3.** Factors Associated With Incidence or Progression: Robust Poisson Regression Results

Measures	Incident DR for patients with no DR at baseline (n = 7097)				Mild NPDR at baseline (n = 1277)	
	Any increase (n = 1280)		2+ step increase (n = 441)		Any increase (n = 347)	
	RR (95% CI)	*P* value	RR (95% CI)	*P* value	RR (95% CI)	*P* value
Intercept	0 (0)	<.001	0 (0)	<.001	22.3 (0-0.1)	<.001
Age, y	1.0 (1.0-1.0)	.09	1.0 (1.0-1.0)	.02	1.0 (1.0-1.0)	. <.001
Sex (self-report)						
Male	1.2 (1.0-1.3)	.004	1.3 (1.1-1.6)	.003	1.1 (0.9-1.3)	.41
Female	1 [Reference]		1 [Reference]		1 [Reference]	
Diabetes duration, y						
<1	0.8 (0.6-1.0)	.03	0.5 (0.3-0.9)	.03	0.9 (0.5-1.8)	.74
1-5	1 [Reference]		1 [Reference]		1 [Reference]	
6-10	1.4 (1.2-1.6)	<.001	2.3 (1.7-3.0)	<.001	1.7 (1.2-2.5)	.004
11-15	1.5 (1.3-1.8)	<.001	2.5 (1.8-3.4)	<.001	2.0 (1.4-2.8)	<.001
>15	2.0 (1.7-2.4)	<.001	3.2 (2.3-4.4)	<.001	1.8 (1.2-2.5)	.002
Not specified	1.4 (1.2-1.7)	<.001	1.7 (1.2-2.6)	.004	1.4 (0.9-2.1)	.13
HbA_1c_, %^a^	1.1 (1.1-1.2)	<.001	1.3 (1.2-1.3)	<.001	1.1 (1.0-1.1)	<.001
Diabetes therapy						
Diet only	1 [Reference]		1 [Reference]		1 [Reference]	
Oral medications only	1.4 (1.1-1.9)	.02	3.0 (1.2-7.3)	.014	1.8 (0.7-4.3)	.20
Insulin only	2.1 (1.5-2.9)	<.001	4.5 (1.8-11.2)	.001	2.3 (0.9-5.5)	.07
Oral medications and insulin	2.2 (1.6-3.0)	<.001	4.5 (1.8-11.1)	.001	2.0 (0.8-4.9)	.12
Not specified	1.5 (1.1-2.0)	.02	3.5 (1.4-8.7)	.008	1.9 (0.8-4.8)	.15
Other patient diagnoses						
Hypercholesterolemia	1.0 (0.9-1.1)	.43	0.7 (0.6-0.9)	.009	0.8 (0.7-1.0)	.03
Cardiovascular disease	0.9 (0.8-1.1)	.51	0.8 (0.6-1.2)	.37	1.1 (0.8-1.5)	.58
Hypertension	1.1 (1.0-1.2)	.13	1.1 (0.9-1.3)	.59	0.9 (0.8-1.1)	.53
Peripheral neuropathy	1.3 (1.1-1.5)	.01	1.1 (0.8-1.6)	.56	1.5 (1.2-2.0)	.001
Nephropathy	1.3 (1.0-1.5)	.03	1.1 (0.8-1.7)	.50	1.2 (0.9-1.7)	.14
Imaging technology						
NMFP						
Both examinations	1 [Reference]		1 [Reference]		1 [Reference]	
First examination, UWFI second	1.5 (1.2-1.9)	<.001	2.2 (1.4-3.5)	.001	1.5 (1.0-2.2)	.05
UWFI						
First examination, NMFP second	0.9 (0.7-1.3)	.74	1.0 (0.5-2.1)	.94	1.1 (0.7-1.7)	.82
Both examinations	1.2 (1.0-1.5)	.04	1.9 (1.2-3.0)	.006	1.1 (0.8-1.6)	.63

Abbreviations: DR, diabetic retinopathy; HbA_1c_, hemoglobin A_1c_; NPDR, nonproliferative DR; NMFP, nonmydriatic fundus photography, RR, risk ratio; UWFI, nonmydriatic ultra-widefield imaging.

SI conversion factor: To convert HbA_1c_ to proportion of total Hb, multiply by 0.01.

^a^ The mean for the sample was substituted for the missing value.

For comparison with other studies (Table 4),^9,10,11,12,21,22,23,24,25,26,27,28,29,30,31^ we conducted several post hoc analyses. These found that of American Indian and Alaska Native patients diagnosed with diabetes before age 30 years and taking insulin alone or with oral medications, 36.9% (90 of 244) developed any new DR within 4 years. Of American Indian and Alaska Native patients diagnosed at 30 years or older and taking insulin, 28.5% (378 of 1324) and 0.1% (1 of 1324) developed any new DR and PDR, respectively. Additionally, a separate regression model with only hypertension and blood pressure medication (yes/no) found that patients with hypertension were 14% more likely to develop new DR and no more or less likely to progress than patients without hypertension.

**Table 4.** Selected Publications on Diabetic Retinopathy (DR) Incidence and/or Progression

Source^a^	Study design	Setting/population	Time frame	Results
**Studies of American Indian or Alaska Native individuals**				
Knowler et al,^9^ 1980	Prospective cohort study (emphasis on blood pressure)	Pima Indians of Arizona, Gila River Indian Community (n = 188 people with diabetes); 77.0% follow-up	1965+, 2 Examinations given, 6 y apart (to within 2 y)	Participants not taking insulin: 24% developed hemorrhages, 24% developed exudates Participants taking insulin: 56% developed hemorrhages, 64% developed exudates
Nelson et al,^10^ 1989	Longitudinal community-based study with medical examinations	Residents of Gila River, Arizona, who were at least 50% Pima and/or Papagado Indian (n = 953); denominator not reported	1983-1987, Biennial examinations	2.6% Incident PDR
Lee et al,^11^ 1992	Cohort follow-up study	IHS clinics serving American Indian individuals within Oklahoma (n = 380); follow-up rate was 41% (total)/73.8% (survivors only)	1972-1980 Baseline, 1987-1991 follow-up	72.3% Incident DR 15.4% Developed PDR
Rith-Najarian et al,^12^ 1993	Analysis of IHS diabetes registry data	Chippewa Indian individuals and related tribes in northern Minnesota, served by an IHS clinic (n = 429); denominator not reported	1986-1988	2.8% Incident PDR (12 cases/1000 PY)
**Studies of Hispanic American individuals**				
Varma et al,^21^ 2010 Population-based, prospective, cohort study (Los Angeles Latino Eye Study)	6 census tracts in La Puente, California; Latino individuals aged ≥40 (n = 775); follow-up rate for people with 2 fundus examinations not reported; 2000-2003 baseline, 2004-2008 follow-up	34.0% Incident DR 38.9% Progressed	14.0% Improved	If NPDR at first examination: 5.3% had PDR at follow-up and 1.9% had PDR with high-risk characteristics
Tudor et al,^22^ 1998	Geographically based case-control study (San Luis Valley Diabetes Study)	Hispanic and non-Hispanic White individuals residing in San Luis Valley of Colorado (n = 244); 62.7% of people examined originally had at least 1 follow-up	1984-1988 Baseline, 1988-1992 follow-up	Incident DR: 26.2% For non-Hispanic White individuals (76.1 cases/1000 PY); 20.8% For Hispanic individuals (58.3 cases/1000 PY) Progression: 27.2% For non-Hispanic White individuals (79.3 cases/1000 PY); 23.0% For Hispanic individuals (65.2 cases/1000 PY)
**Studies predominantly composed of White and Black individuals in the US**				
Klein et al,^23^ 1989	Population-based study (Wisconsin Epidemiologic Study of Diabetic Retinopathy)	People with diabetes who were diagnosed before age 30 y, taking insulin, in southern Wisconsin (n = 891); follow-up rate was 89.5%	1980-1982 Baseline with 1984-1986 follow-up	No DR at baseline: 59.0% Incident DR 0.4% Developed PDR DR at baseline: 10.5% Developed PDR
Klein et al,^24^ 1989	Population-based study (Wisconsin Epidemiologic Study of Diabetic Retinopathy)	People diagnosed with diabetes at age ≥30 y, in southern Wisconsin (n = 987); follow-up rate was 72.0%	July 1, 1979-June 30, 1980, baseline with 1984-1986 follow-up	Not taking insulin: 34.4% Incident DR 2.3% New PDR Taking insulin: 47.4% Incident DR 7.4% New PDR
Wong et al,^25^2007	Population-based, prospective cohort study (Atherosclerosis Risk in Communities Study)	4 communities in the US, dispersed (n = 981 African American and White participants, with and without diabetes, aged 45-64 y); follow-up rate was 90.5%	1993-1995 Baseline with 1996 follow-up	Of those with diabetes: 10.0% incident DR
**International studies**				
Song et al,^26^ 2011	Retrospective study	Hong Kong Chinese individuals enrolled in a diabetic retinopathy screening program, aged ≥30 y (n = 5160); denominator not reported	2005-2009	15.2%, 14.5%, 0.7%, and 0.03% Incident any DR, mild NPDR, moderate NPDR, and sight-threatening retinopathy, respectively 6.6% With baseline DR progressed and 45.5% regressed
Kanjee et al,^27^ 2017	Retrospective chart analysis	49 Communities in Northern Manitoba, Canada (n = 4676; 1976 with no evidence of DR at initial examination and examined at least 2 times); 55.2% follow-up for total sample	May 2007-July 2013	17.1% Incident DR
Burgess et al,^28^ 2017	Cohort study; hospital-based, primary care diabetes clinic	Southern region of Malawi (n = 135); follow-up rate was 53%	2007-2012	48.4% Incident DR % With PDR at follow-up was: 0% Of those with baseline level 10 4.5% Of those with baseline level 20 22% Of those with baseline level 30 40% Of those with baseline level 40
Shani et al,^29^ 2018	Retrospective, longitudinal, community-based study	Israel (n = 516); 67.8% had a first follow-up examination in specified time frame	2000-2002 Baseline, at least 1 follow-up examination by end of 2007	18.8% and 2.7% Incident NPDR and PDR, respectively
				30% Cumulative NPDR (baseline + new incidence)
Li et al,^30^ 2020	Meta-analysis	Europe; 4 studies selected, 2 English and 2 Spanish (n = 71 307 people with type 2 diabetes)	Studies published 1996-2017	4.6% Pooled annual DR incidence calculated by meta-regressions 0.5% Pooled annual sight-threatening DR and/or maculopathy calculated by meta-regressions
Sabanayagam et al,^31^ 2019	Systematic review	International; 8 studies selected, 5 from Asia, and 1 each from North America, Caribbean, and Sub-Saharan Africa	Studies published after 2000	Annual incidence of DR ranged from 2.2% to 12.7% and progression to PDR from 3.4% to 12.3%

Abbreviations: IHS, Indian Health Service; NPDR, nonproliferative DR; PY, person-years at risk.

^a^ Several of these studies examined regression of DR as well, but those results are not enumerated here because regression is not explored in this article.

### UWFI and DR Outcomes

UWFI for follow-up or both examinations was associated with any DR incidence (RR, 1.2; 95% CI, 1.0-1.5; *P* = .04) and a 2+ step increase (RR, 1.9; 95% CI, 1.2-3.0; *P* = .006) in patients with no DR at baseline. For example, compared with patients imaged with NMFP at both examinations, patients imaged with UWFI at follow-up had 2.2 times (95% CI, 1.4-3.5; *P* = .001) the rate of a 2+ step increase in DR (Table 3). UWFI was associated with DR progression when used for the follow-up examination in patients with mild NPDR at baseline.

## Discussion

To prevent or reduce the damaging effects of diabetes complications in American Indian and Alaska Native individuals, the IHS implemented programs such as the Special Diabetes Program for Indians (SPDI) and this American Telemedicine Association Category 3 teleophthalmology program. The SDPI has increased access to diabetes treatment services and reduced hyperglycemia, blood lipid levels, and kidney failure. HbA_1c_ level decreased from 9.0% in 1996 to 8.0% in 2020, low-density lipoprotein cholesterol level decreased from 118 mg/dL in 1998 to 89 mg/dL in 2020 (to convert cholesterol to millimole per liter, multiply by 0.0259), and kidney failure decreased 54% between 1996 and 2013.^32^ The teleophthalmology program itself conducted 264 437 examinations of 120 075 patients between January 1, 2000, and October 31, 2021. Substantial changes in diabetes medications have occurred in the past 25 years as well.^33^ Coinciding with this expansion of diabetes programs and medications, the prevalence of diabetic eye disease in American Indian and Alaska Native individuals served by the IHS teleophthalmology program appears to have declined.^7,8^

This article updates estimates of incidence and progression in this population. Eighteen percent of patients (1280 of 7097) with no evidence of DR in 2015 developed some level of DR during 2016 to 2019, 6.2% (441 of 7097) had a 2+ step increase, and 0.1% (10 of 7097) developed PDR. The estimates reported here are lower than previous estimates in American Indian and Alaska Native patients.^9,10,11,12^ Estimates from this study are also lower than or similar to estimates of DR incidence in Hispanic American individuals.^21,22^

The target populations and methods used in other US-based studies make comparisons to this study problematic.^23,24,25^ For comparison with the benchmark Wisconsin Epidemiologic Study of Diabetic Retinopathy (WESDR),^23,24^ we conducted an additional analysis of DR incidence by age of diabetes diagnosis (before or after 30 years) plus whether the American Indian and Alaska Native patients were taking insulin. The percentages of incident any DR and PDR that we found were lower than those reported in WESDR articles with a similar follow-up period of approximately 4 years.

The estimates from this study are more comparable with studies conducted after 2000 and outside the US.^26,27,29,31^ For example, in Hong Kong, incident any DR was 15.2% and sight-threatening DR was 0.03% in Chinese people surveilled with digital fundus photography.^26^ Approximately 2.2% of patients (28 of 1277) in this study who had mild NPDR at baseline progressed to PDR in the examined time frame compared with 0.1% of patients (10 of 7097) who had no DR at baseline, which is consistent with a recent systematic analysis of international studies.^31^

The influence of most of the risk factors examined on the development of DR was as expected, with duration of diabetes, hyperglycemia, and therapeutic regimen being significant contributors. Additionally, UWFI at the follow-up examination detected more incident DR and more progression of DR, perhaps reflecting the identification of predominantly peripheral lesions (PPL).^34^

The lack of a net effect for hypertension in this study was inconsistent with prior research.^6,9^ A more refined measure of hypertension (such as actual blood pressure measurements) may be needed to understand its association with DR changes in this cohort; however, that information is not currently in the program’s database.

In this national, primary care–based program, UWF fluorescein angiography (UWF-FA) is not available to identify PPL or extent of retinal capillary nonperfusion on FA. Newly published results from the DRCR Retina Network found that, over 4 years, greater initial retinal nonperfusion and FA PPL on UWF-FA were statistically associated with worsening DR as measured by 2+ step progressions or DR treatment.^35^ Based on those findings, more incident DR and DR progression may have been found if UWF-FA was used in this program.

### Strengths and Limitations

A strength of this study includes the geographical distribution of the American Indian and Alaska Native cohort and the large sample size. Previous studies focused on specific areas and had smaller sample sizes. However, the data are exclusively from the IHS, which has a user population of 2.56 million,^36^ representing approximately 25% of the total estimated 9.7 million American Indian and Alaska Native individuals in the US.^37^ Generalizations from this report should be restricted to American Indian and Alaska Native individuals served by the IHS.

There are study limitations to acknowledge. This study focused on DR, omitting DME, another leading cause of vision loss in people with diabetes. Thus, this study likely underestimated the overall burden of diabetic eye disease incidence for American Indian and Alaska Native patients. However, we believe that the underestimate is modest. First, a recent previous report found that DME prevalence in American Indian and Alaska Native patients was 3.0%,^8^ using clinical data from UWFI. Aiello and colleagues^38^ found that UWFI has a low sensitivity for the detection of DME compared with spectral-domain optical coherence tomography, which suggests that DME prevalence estimates derived from UWFI may contain false positives. Second, a recent meta-analysis^30^ of European data showed that annual incidence of DME was 0.4%. Omission of DME in this study may have underestimated the incidence of diabetic eye diseases overall by approximately 1.6% to 2.0%. The percentage might be lower still because some patients with incident DME may have also had incident DR and were already counted in the estimate.

Another potential limitation of this study is that 61.2% of the total patients evaluated by the program in the baseline period had an examination during the follow-up period; ie, 38.8% were not reexamined in 2016 to 2019. This follow-up rate is lower than several studies,^9,23,24,25^ but similar to^22^ or higher than^11,28^ other studies. Some retrospective studies do not report a denominator, precluding comparison of this study’s follow-up rate with theirs. To understand the implications of 38.8% attrition for the results, we compared the baseline characteristics of patients who were not reexamined with those who were and found that the groups were similar except that the followed cohort was slightly less healthy. The estimates reported here likely are reasonable even with the attrition rate.

## Conclusions

The results of this cohort study suggest that recent DR incidence and progression among American Indian and Alaska Native individuals served by the IHS are substantially lower than they were 30 or more years ago and are now comparable with estimates from non–American Indian and Alaska Native populations examined in the last 20 years. Further, these low rates support the viability of safely extending the follow-up interval for retinopathy assessment in IHS patients who have no evidence of DR or mild NPDR. This may be possible if the IHS patients also have no DME, have minimal risk factors, will be examined with UWFI, and their follow-up adherence is not jeopardized. Currently, the IHS teleophthalmology program recommends annual DR examinations, consistent with the American Academy of Ophthalmology Preferred Practice Patterns^39^ and those of other professional organizations, but biennial frequency as recommended by the American Diabetes Association^40^ is well documented and might be an appropriate frequency for the IHS as well. Such a practice change, however, requires examination of adherence to the current recommendations. If a practice change extending follow-up were implemented, further research would be needed to determine if the change affected vision outcomes and adherence rates.

## Notes

Supplement. Data Sharing Statement
