# Supplementary material for: Incidence and Progression of Diabetic Retinopathy in American Indian and Alaska Native Individuals Served by the Indian Health Service, 2015-2019
Source: JAMA Ophthalmol. 2023 Mar 9;141(4):366–75. doi: 10.1001/jamaophthalmol.2023.0167 (PMC9999279; doi:10.1001/jamaophthalmol.2023.0167)
Supplement: Supplement. — Data Sharing Statement [file jamaophthalmol-e230167-s001.pdf]

Data Sharing Statement

Data

Data available: No

Additional Information

Explanation for why data not available: Generally, the Indian Health Service (which is the setting for this study) does not share patient datasets. This is due to mistrust and concern within the population it serves -- about research in general -- which in turn is due to history. A special exemption would need to be granted. At the moment, the IRB has not approved data sharing for this project.
